# Epithelial cell adhesion efficacy of a novel peptide identified by panning on a smooth titanium surface

**DOI:** 10.1038/s41368-018-0022-1

**Published:** 2018-07-02

**Authors:** Hidemichi Kihara, David M. Kim, Masazumi Nagai, Toshiki Nojiri, Shigemi Nagai, Chia-Yu Chen, Cliff Lee, Wataru Hatakeyama, Hisatomo Kondo, John Da Silva

**Affiliations:** 1 Harvard School of Dental Medicine, Boston, MA USA; 2 School of Dental Medicine, Iwate Medical University, Iwate, Japan; 3 University of California, San Francisco, CA USA

## Abstract

Epithelial attachment via the basal lamina on the tooth surface provides an important structural defence mechanism against bacterial invasion in combating periodontal disease. However, when considering dental implants, strong epithelial attachment does not exist throughout the titanium-soft tissue interface, making soft tissues more susceptible to peri-implant disease. This study introduced a novel synthetic peptide (A10) to enhance epithelial attachment. A10 was identified from a bacterial peptide display library and synthesized. A10 and protease-activated receptor 4-activating peptide (PAR4-AP, positive control) were immobilized on commercially pure titanium. The peptide-treated titanium showed high epithelial cell migration ability during incubation in platelet-rich plasma. We confirmed the development of dense and expanded BL (stained by Ln5) with pericellular junctions (stained by ZO1) on the peptide-treated titanium surface. In an adhesion assay of epithelial cells on A10-treated titanium, PAR4-AP-treated titanium, bovine root and non-treated titanium, A10-treated titanium and PAR4-AP-treated titanium showed significantly stronger adhesion than non-treated titanium. PAR4-AP-treated titanium showed significantly higher inflammatory cytokine release than non-treated titanium. There was no significant difference in inflammatory cytokine release between A10-treated and non-treated titanium. These results indicated that A10 could induce the adhesion and migration of epithelial cells with low inflammatory cytokine release. This novel peptide has a potentially useful application that could improve clinical outcomes with titanium implants and abutments by reducing or preventing peri-implant disease.

## Introduction

The structure and mechanism of epithelial attachment around dental implants and abutments are different from those around natural teeth.^1^ Instead of the tight epithelial sealing on tooth root surfaces seen in natural dentition, there is poor attachment with few basal lamina (BL) on the titanium surface in the transmucosal area.^2–5^ Poor epithelial attachment can lead to bacterial invasion and colonization, causing soft tissue inflammation and alveolar bone resorption in a process similar to periodontal disease. The clinical success rate of dental implants reported since 2010 varies from 51.9% to 95.9%,^6–10^ which is partly due to variations in the criteria for success used in these studies. Peri-implantitis is a significant complication of dental implants that is caused by bacterial infection; it is difficult to predictably treat and can lead to implant failure.^11,12^ To prevent peri-implantitis and increase dental implant success rates, it is critical to establish a strong epithelial seal at the implant/abutment surface in the cervical region.

Numerous efforts have been made to achieve strong soft tissue attachment to the titanium surface to preserve soft and hard tissue support. Studies have shown that rough titanium surfaces and/or grooved titanium surfaces have better soft tissue attachment,^13,14^ but they also have increased susceptibility to bacterial plaque formation, leading to more inflammation than smooth surface implants.^15^ To achieve a successful long-term prognosis, the establishment of strong epithelial attachment and a strong seal on the smooth titanium surface of the implant/abutment is important.

Sugawara et al.^16^ reported the establishment of BL-mediated epithelial cell attachment to the treated smooth titanium surface through the use of platelets. The platelets were activated on the smooth titanium surface using protease-activated receptor-4 activating peptide (PAR4-AP) to create an altered surface that promoted epithelial binding. For the first time, it was found that PAR4-AP stimulated the release of chemokines and growth factors, including epidermal growth factor (EGF) and insulin-like growth factor 1 (IGF-1), from the activated platelet aggregate. Our previous study indicated that the platelet-induced epithelial sheet on PAR4-AP-treated titanium blocked bacterial invasion.^17^ This result indicated the establishment of strong epithelial adhesion on the smooth titanium surface, but the adhesion strength could not be quantified. To date, rotary shakers and gentle washes have been used to assess epithelial adhesion strength;^14,18,19^ however, these methods generate fluid shear stress, which cannot be expressed as an immutable unit of measure. In some situations, PAR4 agonists have also been shown to induce leukocyte rolling,^20,21^ and this result may suggest that the receptor has a proinflammatory role in vivo. To identify a peptide that results in reduced inflammatory cytokine release and stronger epithelial cell adhesion than PAR4-AP, the isolation and identification of peptides involved in the induction of epithelial cells is necessary so that these peptides can be further studied. Cell panning is a powerful tool used to select affinity peptides on immobilized target molecules.^22^ We combined the mechanisms of platelet activation with cell panning in order to identify novel peptides that not only have binding affinity but also cause platelet aggregation to stimulate chemokines and growth factors and establish strong epithelial attachment.

In this study, we introduced novel peptides selected from a bacterial peptide library that promoted increased chemokine and growth factor stimulation. Epithelial cell migration from surface-treated titanium with novel peptides was compared to migration from PAR4-AP-treated titanium and untreated titanium, and the adhesion strength of epithelial attachment was measured quantitatively using centrifugal force.

## Results

**Fig. 1:**
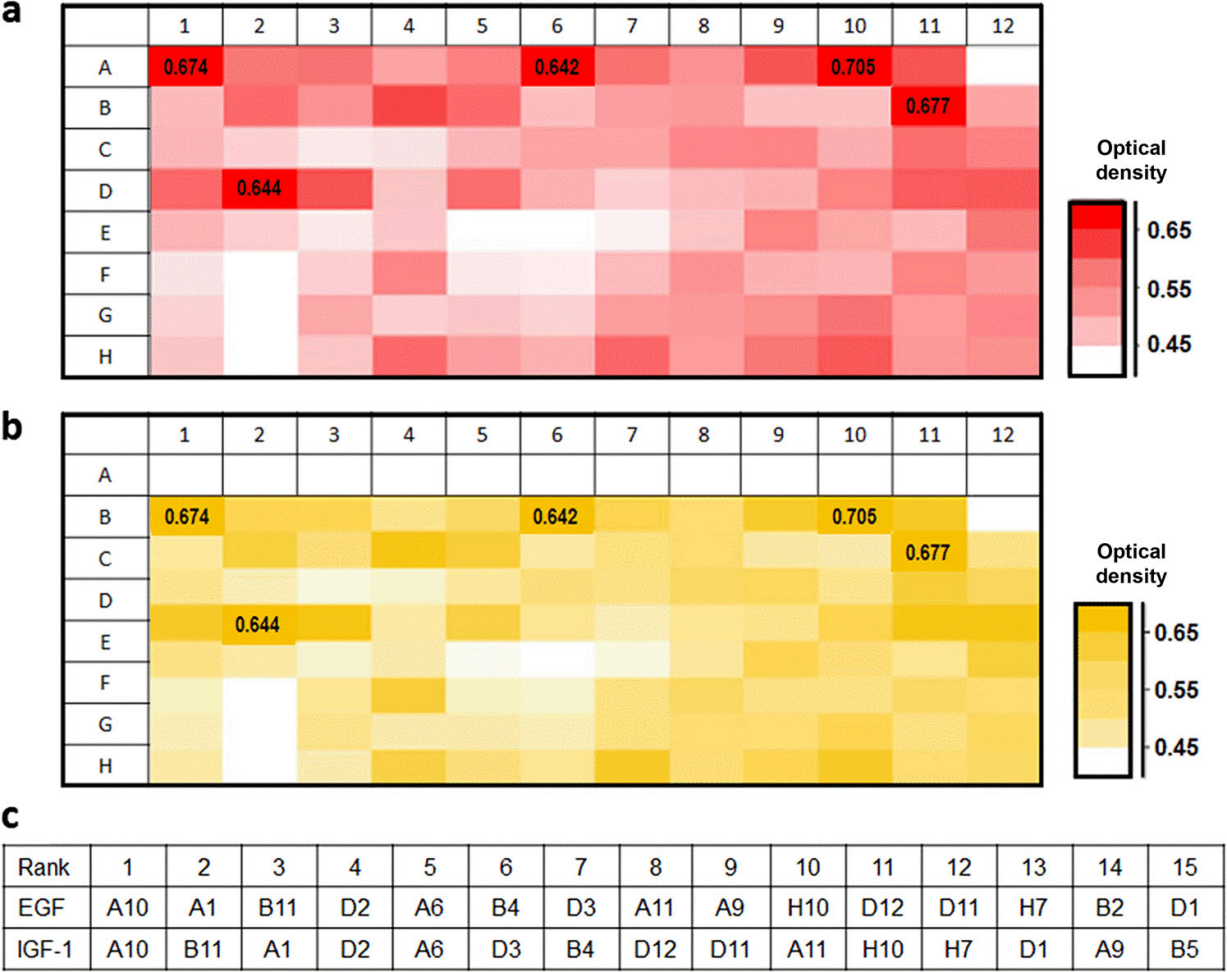
Flagellin peptide-stimulated release of EGF and IGF-1 from PRP on the treated titanium surfaces. Heat maps express the optical densities of ELISA on **a** EGF and **b** IGF-1. The top 15 peptides that stimulated release are shown in **c**

**Fig. 2:**
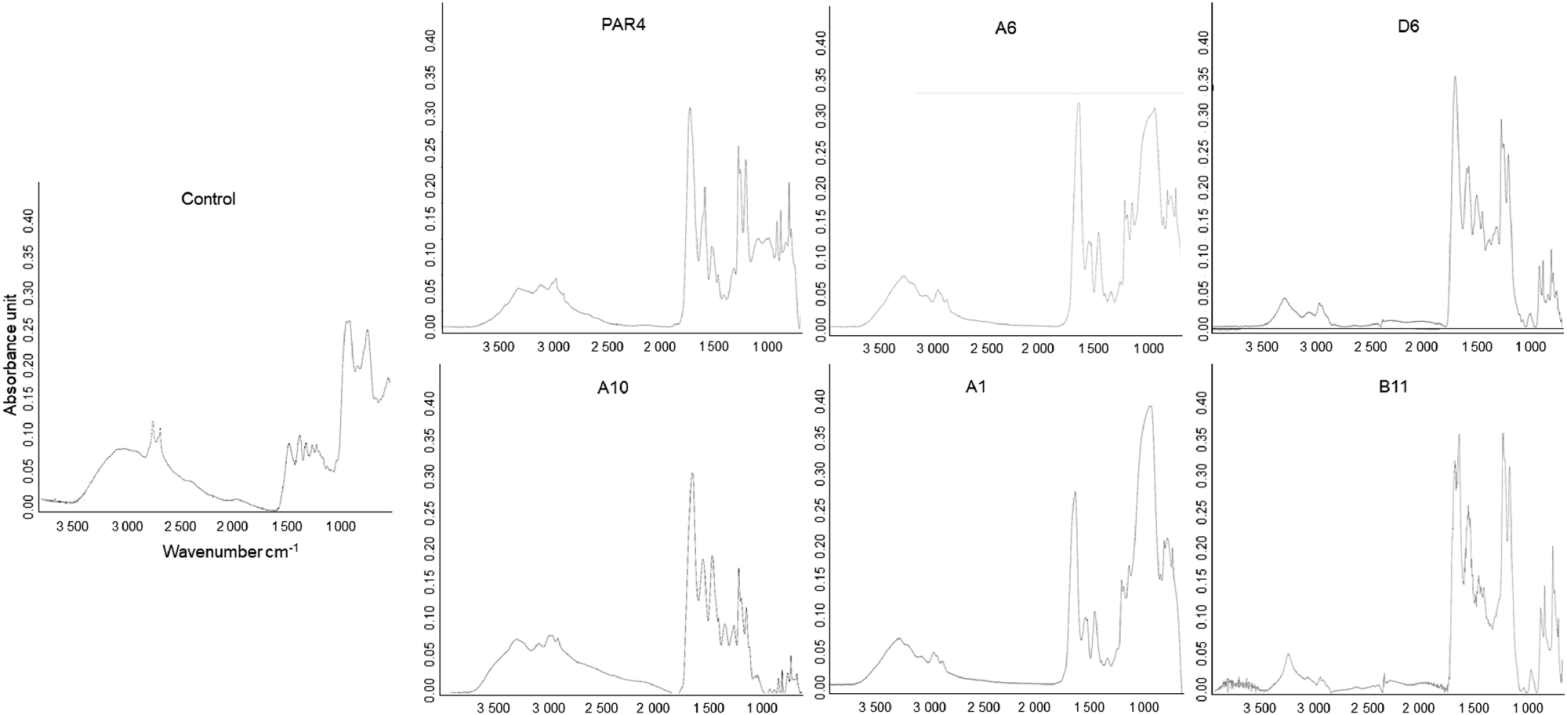
FTIR spectra of linker-derived and peptide-derived peaks on titanium. Spectra of titanium treated with CDPA linker is shown as “Control” in Fig. 2. Five peptide-derived peaks of amide I (~1 650 cm^−1^) and amide II (~1 540 cm^−1^) were detected on the treated titanium surface with five peptides, indicating the immobilization of peptides on the titanium surface

Identification of platelet-activating peptides based on the activities of epithelial chemokine and growth factor induction. The induction of EGF (Fig. 1a) and IGF-1 (Fig. 1b) by 96 peptides identified in the primary screening was compared in a heat map. The top 5 peptides for EGF and IGF-1 induction included A10, A1, B11, D2, and A6 (Fig. 1c). The DNA sequences encoding these five peptides were determined in their corresponding bacteria (Table 1). The five peptides were then synthesized for testing on the titanium surface and immobilized on the smooth titanium surface via a CDPA linker. Immobilization of the five peptides on the treated titanium surface was verified by the spectrum peaks of amide I (~1 650 cm^−1^) and amide II (~1 540 cm^−1^) as observed on Fourier transform infrared spectroscopy (FTIR) (Fig. 2). Titanium that was not treated with peptides was verified by a lack of spectrum peaks for the amide groups (Fig. 2: control).

**Table 1:** Amino acid sequences and corresponding DNA sequences

**A1**: CGPAEKAYPNNSPLFGPC
TGCGGCCCAGCGGAAAAGGCTTATCCAAATAATTCCCCGCTTTTTGGCCCCTGT
**A10**: CGPPPGNPKIKWPPGGPC
TGTGGACCGCCACCAGGGAATCCGAAAATCAAATGGCCGCCCGGTGGCCCGTGT
**A6**: CGPYIPQPRPPGPRLGPC
TGTGGTCCTTACATTCCACAGCCCCGTCCTCCCGGCCCCCGTTTAGGACCATGT
**B11**: CGPTGRYTIHTLLRFGPC
TGCGGACCGACAGGTCGTTATACCATCCATACTCTGCTGCGTTTTGGACCCTGT
**D2**: CGPLFLLRNGYPGKFGPC
TGCGGTCCCCTGTTCTTGTTGCGTAACGGTTACCCTGGTAAATTCGGCCCGTGC

Unique 12mer amino acid sequence was flanked between N-term (CGP) and C-term (GPC) of which cysteines formed disulfide bond

**Fig. 3:**
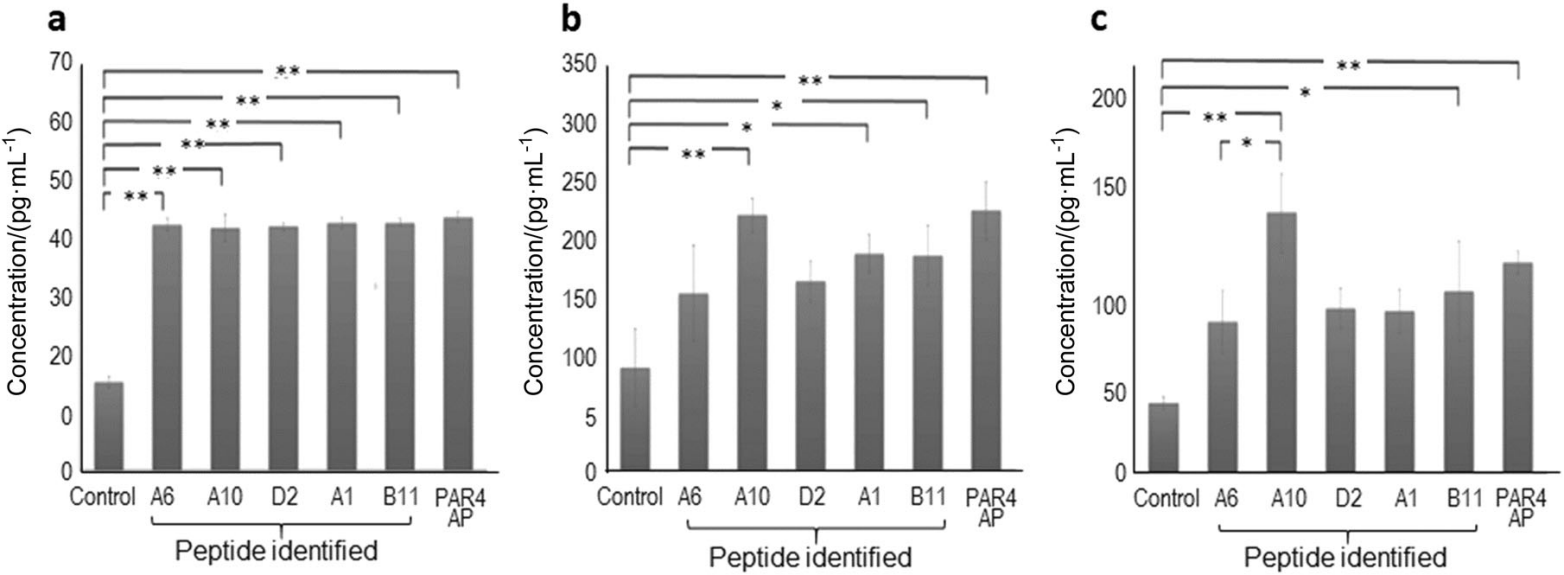
Platelet-released EGF, IGF-I, and TGF-beta on the treated titanium surfaces with synthetic peptides. Bars indicate the concentrations of **a** EGF, **b** IGF-I, and **c** TGF-beta in the platelet-rich plasma (PRP) supernatant. Values are the means ± standard deviations (*n* = 3). The data were analysed with one-way ANOVA and Scheffé post hoc test (*P < 0.05, **P < 0.01)

PAR4-AP-treated titanium and all five synthetic peptide-treated titanium samples released significantly higher EGF than non-treated titanium (Fig. 3a; *P* < 0.01). A10-treated, A1-treated, B11-treated, and PAR4-AP-treated titanium released significantly higher IGF-1 than non-treated titanium (Fig. 3b; A10 and PAR4-AP: *P* < 0.01, A1 and B11: *P* < 0.05). The IGF-I release from A6-treated titanium and D2-treated titanium was not significantly higher than the IGF-I release from the control. EGF, IGF-1, and TGF release was significantly higher from A10-treated titanium and B11-treated titanium than from the control. Moreover, IGF-1 and TGF-B release was higher from A10-treated titanium than from B11-treated titanium. Finally, we selected A10 and conducted the following experiments.

### Evaluation of function of novel peptide A10

#### Inflammatory cytokine release

**Fig. 4:**
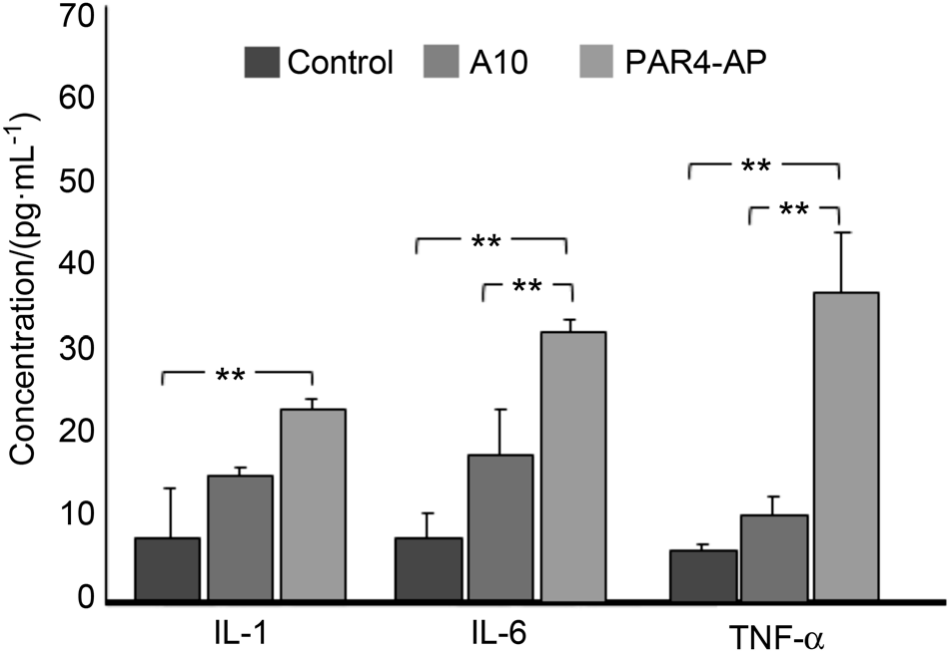
Inflammatory cytokine release from whole blood on the treated titanium surfaces with synthetic peptides. The concentration unit is pg·mL^−1^ in the supernatant of whole blood. Values are means ± standard deviations (*n* = 3). The data were analysed with one-way ANOVA and Scheffé post hoc test (**P < 0.01). There is no significant difference (*P* > 0.05) between A10-treated titanium and the smooth titanium surface (control) for any marker

PAR4-AP-treated titanium released significantly higher levels of interleukin (IL)-1β, IL-6, and TNF-α than the control (*P* < 0.01). In contrast, there was no significant difference between A10-treated titanium and the control for IL-1β, IL-6, and tumour necrosis factor (TNF)-α release (Fig. 4).

#### Epithelial cell migration and attachment

**Fig. 5:**
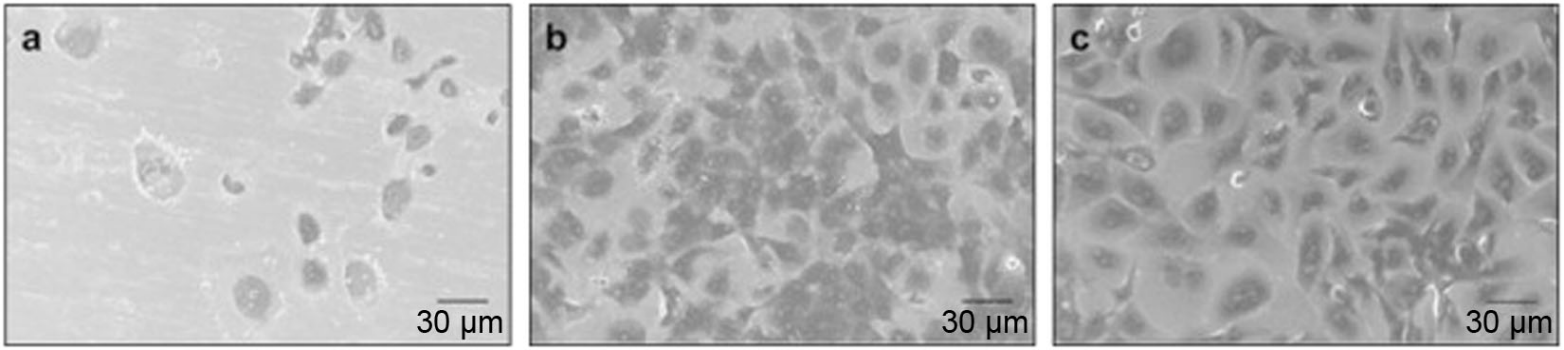
Scanning electron microscopy (SEM) ultrastructure of epithelial cells on the peptide-treated titanium surfaces. Representative SEM micrographs at 1000× magnification of OBA9 epithelial cells for the **a** control and **b** A10-treated and **c** PAR4-AP-treated titanium surfaces

**Fig. 6:**
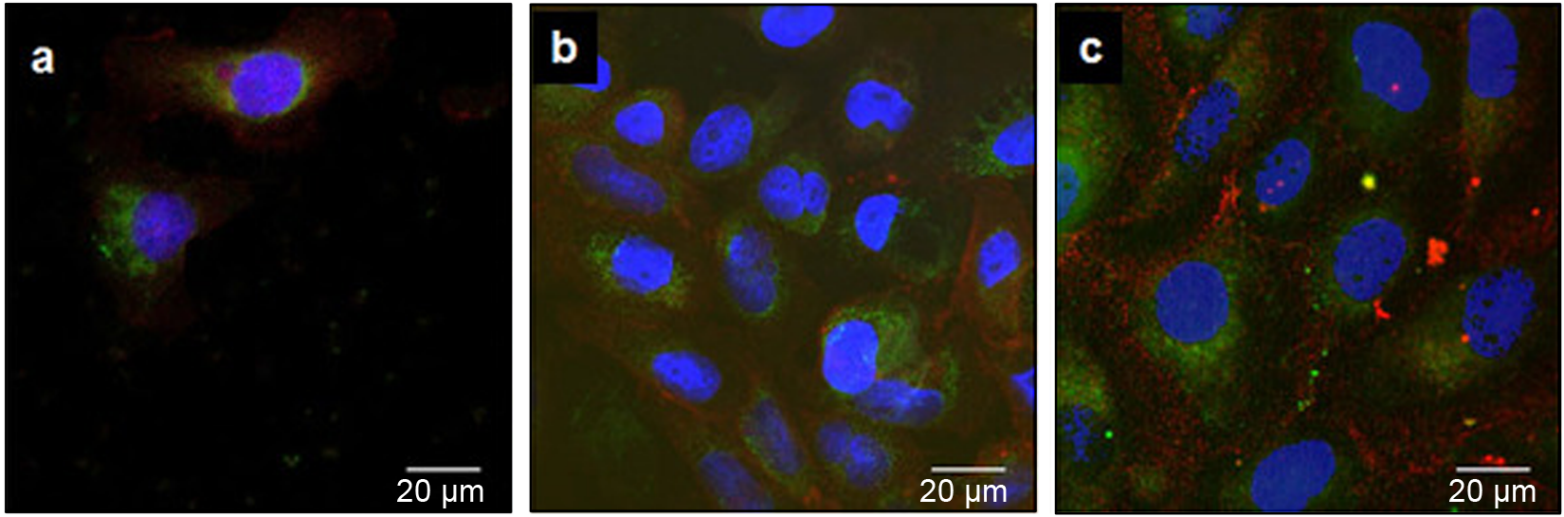
Immunocytochemical staining of BL component proteins in OBA9 epithelial cells on the treated titanium surfaces with synthetic peptides. Representative confocal micrographs of Ln5 (green) and ZO1 (red) on the **a** control, **b** A10-treated, and **c** PAR4-AP-treated titanium surfaces. Nuclei were counter-stained with DAPI (blue)

Dense epithelial cells were observed on the titanium surfaces treated with A10 and PAR4-AP (Fig. 5b,c), whereas few cells were seen on the non-treated smooth titanium surface (Fig. 5a). This result indicated that the cells actively transmigrated from the culture insert to the treated surfaces through the narrow pore. Confocal images confirmed many cell nuclei counter-stained with DAPI on the surfaces treated with A10 and PAR4-AP (Fig. 6b,c). The development of dense and expanded BL (stained by Ln5) with pericellular junctions (stained by ZO1) was found on the treated titanium surfaces (Fig. 6b,c). In contrast, low cell migration was observed on the non-treated surface (control, Fig. 6a).

#### Epithelial cell adhesion strength

**Fig. 7:**
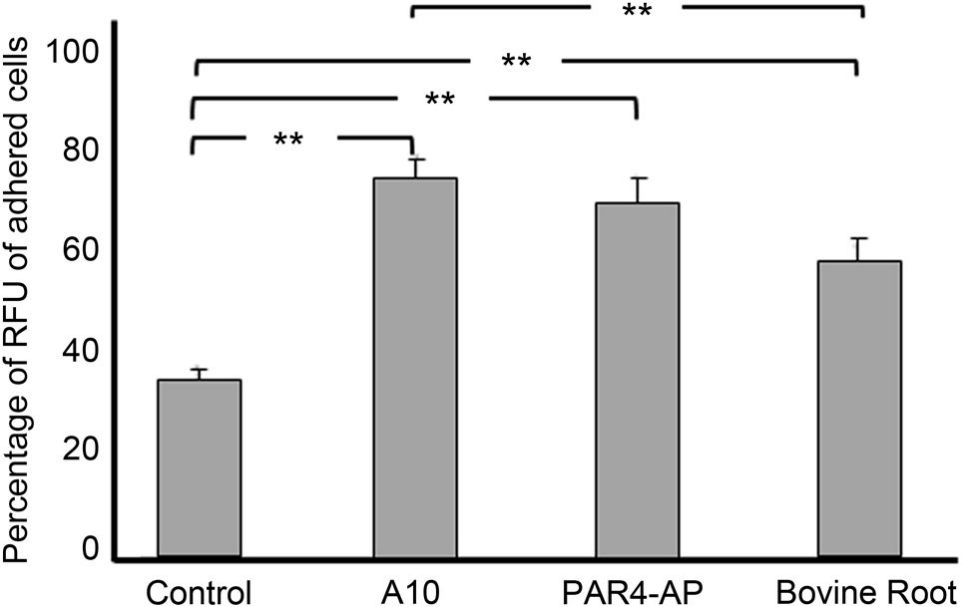
Platelet-promoted adhesion of OBA9 epithelial cells on bovine root and the titanium surfaces treated with A10 and PAR4-AP peptides. Bars indicate the percentage of cells remaining on the treated titanium and bovine root surfaces after centrifugation at 350 × *g*. Values are means ± standard deviations (*n* = 3). Data were analysed with one-way ANOVA and Scheffé post hoc test (**P < 0.01)

There was a significant difference (*P* < 0.01) in the percentage of adherent cells on the non-treated titanium surface (negative control) versus bovine root (positive control) (Fig. 7). The A10-treated surface had the highest percentage of adherent cells compared to the other groups (*P* < 0.01). PAR4-AP-treated titanium had a significantly higher percentage of adherent cells than the control, but there was no significant difference between the percentage of adherent cells in PAR4-AP-treated titanium and bovine root (Fig. 7, *P* < 0.01).

## Discussion

### Significance of platelet-activating titanium surface for dental implants

Because platelets contain various wound healing factors,^24^ the application of platelet-rich plasma (PRP) over wound sites, including surgical incisions, is becoming a common therapeutic modality. However, simply applying PRP to an implant/abutment surface and allowing wound healing to occur has not been shown to establish epithelial BL attachment. A fundamental cause of failure in establishing epithelial BL attachment is that the smooth titanium surface of the implant neck area has no functional molecular groups equipped for platelet activation and fibrin-clot aggregation, which are necessary for wound healing. This characteristic may be desirable in many biomedical applications for avoiding biomaterial-associated thrombosis. However, thrombosis-related morbidity is not relevant to dental implants. We intend to activate platelets for physiological hemostasis, which recruits not only gingival epithelial and connective tissue cells for wound healing but also immune cells to battle microbial infection.

### Identification of novel functional peptides

Our previous study employed PAR4-AP to treat titanium for the activation and aggregation of platelets to induce their robust wound healing potential. As anticipated, platelets rapidly formed aggregates on the PAR4-AP-treated titanium surface that released multiple chemokines and growth factors, yielding an antibacterial epithelial seal with BL attachment.^16,17^ These studies proved that titanium can be treated to enable strong epithelial attachment. To isolate an advanced peptide with improved epithelial sealing but without undesirable side effects, we conducted a novel panning study on whole-cell platelets. While conventional panning only focuses on the binding affinity between peptides and immobilized target molecules, our panning enabled serial screenings following primary aggregation-triggered platelet factor secretion by ELISA in a 96-well format. While the present study identified coagulant peptides, our primary screening can also be used to select anticoagulant peptides in negative selection mode in the presence of hemostatic agents, such as ADP, serotonin or TXB2.

The five novel peptides identified in the present study showed comparable or better effects than PAR4-AP in terms of the induction of epithelial chemokines and growth factors. All five peptides and PAR4-AP had similarly high levels of EGF release. This release was not due to saturation by excessive protein concentrations because the absorbance values were all within the linear section of standard curves. A considerable advantage that the novel A10 peptide had over PAR4-AP was a significantly lower level of induction of inflammatory cytokines. PAR4-AP has been reported to cause inflammation.^21,25–31^ It has been reported that PARs were seen on the inflammatory cell surface.^32–38^ PAR4 was also expressed in mast cells, monocytes, macrophages, and T cells.^32–34,37,38^ Furthermore, Suo et al.^39^ reported that PAR4-AP promoted NFκB activation through the signalling pathway of p44/42 MAPKs and then induced the production of inflammatory cytokines. Since bone resorption in periodontal disease and peri-implantitis have been shown to be largely due to the inflammatory host response,^40^ the induction of inflammatory cytokines by PAR4-AP could have this unintended side effect clinically. To exclude potential drawbacks, we evaluated the effect of the synthetic peptides on inflammatory cytokine release. Our novel peptide A10 demonstrated significantly less release of cytokines IL-1 and IL-6 than PAR4-AP, and there was no significant difference in the release of cytokines IL-1, IL-6, and TNF-α between the A10-treated titanium and the non-treated smooth titanium. The A10 peptide induced growth factors and epithelial chemokines with low inflammatory cytokine release.

### Active migration of epithelial cells

Many of the titanium surface modifications that have been designed to improve epithelial adhesion have been evaluated in cell culture.^14,18,19^ However, these assays did not reflect the in vivo environment of epithelial cells at a surgical wound. The conventional cell adhesion assay includes two significant differences from the in vivo situation. The first difference is the use of a dispersed single cell suspension. Epithelial cells do not exist freely in suspension as they are always linked to adjacent cells with desmosomes, except for during mitosis. The second difference is passive gravity-mediated sedimentation. Since cells passively settle onto the test surface by gravity, the chemotactic activity of the treated surface cannot be accurately judged in the adhesion assay. Furthermore, because the epithelial cell nucleus, which is the heaviest organelle in the cell, tends to locate on the BL side, the epithelial cells passively settle onto the titanium surface with BL due to gravity rather than by migrating and attaching with the correct orientation and BL apparatus. Thus, the active induction of BL attachment to the treated surface cannot be accurately evaluated in this manner. To measure active epithelial migration, we used a Boyden type culture insert with an 8-µm pore mesh that epithelial cells cannot passively move through. The strong epithelial cell migration observed on treated titanium surfaces (Figs. 5 and 6) is a result of cell amoeboid movement due to the concentration gradient of EGF, IGF-1, and TGF-beta released from activated platelets.

### Adhesion strength of epithelial cell attachment

In this study, we established new assay utilizing centrifugation to quantify the adhesive strength of epithelial cells on a smooth titanium surface. Previous in vitro studies testing epithelial cell adhesion used the fluid shear stress generated by gentle washing or a rotary shaker and/or histological analysis to evaluate adhesion strength.^14,18,19^ Those methods cannot standardize the force and/or interpret the forces on the translational unit, such as the force of gravity. The method used in this study employed centrifugal force, which is an immutable unit of measure.

To assess epithelial cell adhesion strength on our treated titanium surface, bovine root was used as a positive control. No studies were found that used natural teeth as a comparison. The titanium surface treated with the novel peptide A10 showed significantly higher cell adhesion not only compared to the non-treated surface but also compared to the bovine root surface, suggesting its promising clinical relevance. One possible explanation of this strong epithelial cell adhesion could be the type IV collagen deposited by platelets that were activated by the peptide.^17^ Type IV collagen is an extracellular matrix that becomes an anchor of integrin penetration from inside the cell. This finding indicates that activated platelets not only induced the migration of epithelial cells to the titanium surface but also created an adhesive scaffold. Another contributing factor could be strong peptide immobilization. For peptide immobilization, we utilized a phosphonic acid linker that binds stably to the titanium surface via bidentate or tridentate.^41^ Therefore, it could be interpreted that the deposited type IV collagen strongly binds onto the titanium surface via a peptide-phosphonic acid linker. A concern arises about potential bacteria colonization on the peptide-linker surface. However, this scenario is unlikely as our previous study confirmed that there was no significant difference in bacterial adhesion between a smooth non-treated titanium surface and a phosphonic acid linker-PAR4-AP surface.^17^

In this study, we established primary strong epithelial adhesion on our treated titanium surface in vitro with a low inflammatory cytokine response. However, the inflammatory phase is the first step in wound healing.^42^ Therefore, the quantitative kinetics of inflammation and healing promotion of cytokines on the respective relevant physiologic and pathological responses of peri-implant tissues should be addressed in future studies. For a successful implant restoration, the specific mechanisms of action of the peptide, such as signalling pathways and effectors, remain to be discovered. Continuous epithelial adhesion in daughter epithelial cells needs to be assessed in an in vivo setting.

In conclusion, we successfully isolated and identified a novel peptide that promoted platelet activation to induce wound healing by promoting IGF-I and TGF-β. While these preferable cytokine inductions were as high as those of the positive control (PAR4-AP), the A10 peptide induced lower levels of inflammatory cytokines IL-1, IL-6, and TNFα than PAR4-AP. As a result, the A10- and PAR4-AP-treated titanium surfaces allowed stronger epithelial attachment than the untreated titanium and bovine root control surfaces. Although A10-induced epithelial attachment was not significantly greater than PAR4-AP-induced attachment, the lower induction of inflammatory cytokines suggests an advanced effect of the A10 peptide.

## Materials and methods

### Whole-cell panning for the identification of novel peptides

#### Primary screening

A bacterial peptide display library (Flitrx, Invitrogen; Thermo Fischer Scientific, MA, USA) was used for the panning of platelet aggregate-activating peptides. The library was composed of 280 million collections of random dodecamer peptides that were displayed on the N-term end of flagellin in bacterial cell membranes. Peptide-displaying bacteria were incubated with human PRP (8.3 × 10^5^ platelets per µL) at 5 × 10^8^ bacterial cfu per 6.25 × 10^8^ platelets for 30 min to isolate the bacterial peptides that stimulated platelet aggregation. PRP was prepared by the centrifugation of citrate-anticoagulated human whole blood (BioreclamationIVT, NY, USA) at 180 × *g* for 15 min at room temperature.

The incubation was applied in a cell strainer with a 100-µm mesh membrane (Biologix, Shandong, China) to capture the platelet aggregate. The aggregate was washed twice with PBS to remove free platelets and bacteria. Then, the bound bacterial peptides were amplified for the next round of panning. The panning step was repeated for three rounds.

The final panned peptide-displaying bacteria were streaked on agar plates to isolate clones. Ninety-six bacterial peptide clones were amplified in liquid culture in the 96-well plate, and fragile flagellin from host bacterial cells was cleaved off by vigorous vortexing. Bacteria-free flagellin peptides were evaluated for the induction of epithelial chemokines and growth factors from platelets in the secondary screening.

#### Secondary screening

Approximately 25 µL of PRP was incubated with 25 µL of cleaved flagellin peptide supernatant from the 96 clones, and the PRP peptide supernatant was analysed for EGF and IGF-1 using ELISA kits (R&D Systems, MN, USA) on a plate reader (FilterMax F5; Molecular Devices, CA, USA). The top 5 clones, which were ranked according to the induction of EGF and IGF-1, were sequenced to obtain the peptide-coding DNA. The five peptides were then chemically synthesized (GenScript, NJ, USA).

### Validation of platelet activation by the synthetic peptides immobilized on the smooth titanium surface

#### Immobilization of peptides and confirmation by FTIR

Five synthetic peptides (A1, A10, A6, B11, and D2) and PAR4-AP (AYPGKF-NH2, positive control; Peptides International, KY, USA) were immobilized on the titanium surface (*n* = 3 for each group). First, commercially pure titanium foil (GalliumSource, LLC, CA, USA, 0.05-mm thick) was cut into 10 mm × 10 mm square pieces, and the titanium pieces were ultrasonically cleaned in 0.5% sodium dodecyl sulfate (SDS; Sigma, MO, USA), deionized water, acetone (Sigma) and ethanol (Sigma) sequentially for 20 min in each solution. The clean titanium pieces were incubated for 3 h in 1 mmol·L^−1^ phosphonic acid linker with a carboxyl end (10-carboxydecylphosphonic acid: CDPA linker, Dojindo Molecular Technology, MD, USA) in ethanol at 70 °C. The titanium pieces were washed in ethanol four times, air dried, and heated to 120 °C for 24 h in a drying oven. The carboxyl residue of the linker was activated by rotating the titanium pieces at room temperature for 2 h in 0.2 mol·L^−1^ N-hydroxysuccinimide (NHS; Sigma) and 0.25 mol·L^−1^ 1-ethyl-3-(3-dimethylaminopropyl) carbodiimide (EDC; Sigma) dissolved in dimethylformamide (DMF; Sigma). After washing four times in DMF, the linker was coupled with the five synthesized peptides and PAR4-AP by rotating the titanium pieces at room temperature for 70 min in DMF containing 0.1 mmol·L^−1^ peptide. The titanium pieces were then washed four times with DMF, air dried, and stored at 4 °C.

Immobilization of the peptides was verified by FTIR. The treated titanium pieces were examined by FTIR (LUMOS; BRUKER, MA, USA) with the wavenumber set to a range of 400–4 000 cm^−1^. The spectrum of a peptide is dominated by its backbone amide I (C=O stretching: near 1 650 cm^−1^) and amide II (C–N stretching, NH bend: 1 550 cm^−1^) vibrations^23^. The peptide-derived peaks from the amide groups were monitored on the treated titanium.

#### Evaluation of growth factor release from platelet aggregate

PRP was inoculated on non-treated, PAR4-AP treated, and five synthetic peptide-treated titanium pieces in a 24-well format (*n* = 3 for each group). Culture supernatant was collected from PRP culture in 24-well format after an incubation time of 180 min. EGF, IGF-1, and TGF-β in the supernatant was measured using ELISA kits (R&D Systems, MN, USA) for peptide selection. The peptide with the best result from ELISA was applied in the next experiment.

### Evaluation of inflammatory cytokine release

The effects of the peptide-treated titanium surface on the release of inflammatory cytokines was evaluated in whole blood (*n* = 3 for each group). Approximately 250 µL of human whole blood (BioreclamationIVT, NY, USA) was applied onto a titanium piece and incubated for 180 min in a 24-well plate (ultra-low attachment plate; Corning, NY, USA). IL-1β, IL-6, and TNF-α were measured by corresponding ELISA kits (R&D Systems).

### Evaluation of epithelial cell migration, attachment, and adhesion strength

#### Epithelial migration and attachment

The treated titanium pieces with the selected peptide and PAR4-AP (*n* = 3 for each group) were cultured in 500 µL of PRP in a 24-well plate (ultra-low attachment plate; Corning) for 180 min, gently washed with phosphate buffer solution (PBS), and immersed in keratinocyte-serum free medium (SFM; Thermo Fischer Scientific) without supplements, including EGF. A Boyden type culture insert with an 8-µm pore filter (Corning) was placed over the titanium piece. Human gingiva-derived epithelial cells (OBA9) were seeded at 1 × 10^5^ cells in the culture insert. After 72 h of incubation, the titanium pieces were washed twice with PBS and fixed in 4% paraformaldehyde for ultrastructure scanning electron microscopy (SEM; Zeiss Supra 55 VP field emission scanning electron microscope; ZEISS, Oberkochen, Germany) and immunocytochemistry by confocal laser microscope (Zeiss LSM 710 confocal microscope; ZEISS); laminin5 (Ln5), a major BL component, and ZO1, a protein found in epithelial cell tight junctions, were stained with fluorescence-conjugated antibodies (Ln5 antibody, DyLight 488 conjugate, ZO1 antibody, Alexa Fluor 594 conjugate; Thermo Fisher Scientific). Cell nuclei were counter-stained with DAPI (FluoroshieldTM with DAPI; Sigma) and imaged under confocal microscopy.

#### Strength of epithelial cell adhesion

Centrifugation was used to measure the strength of epithelial cell adhesion. Bovine roots were cut into 8 mm × 7 mm × 1 mm rectangles and used as a positive control. The titanium with and without peptide and bovine root pieces (*n* = 3 for each group) were incubated in PRP in a 24-well plate (ultra-low attachment plate; Corning) for 180 min, gently washed with PBS, and immersed in 500 µL of supplement-free keratinocyte-SFM. OBA9 cells were seeded at 3 × 10^5^ cells in each well. After 72 h of incubation, each titanium and root piece were placed in 1.0 mL of PBS in a microtube. The microtubes were centrifuged at 350 × *g* for 2 min at room temperature. After centrifugation, detached cells in PBS were transferred to a new microtube, and the remaining cells on each titanium and bovine root piece were detached by enzymatic digestion in 50 μL of trypsin-EDTA (TrypLe select, Thermo Fisher Scientific) for 5 min. These cells were centrifuged and stained in 100 μL of DNA-dye solution containing 2.5 μg·mL^−1^ of Hoechst 33342 (Thermo Fisher Scientific) in 4% PFA-0.2% Triton. After staining for 10 min, cells were centrifuged and washed with PBS three times, and the stained cells were transferred to a 96-well black opaque plate. The relative fluorescence units (RFU) of DNA fluorescence were measured at 360 nm/excitation and 430 nm/emission on a plate reader. The percentage RFU of cells that remained on the testing materials was calculated.

### Statistical analysis

All quantitative data points represent the mean ± standard deviations (SD). Statistical significance was determined by one-way ANOVA and Scheffé post hoc test. Significance levels were determined a priori for *P < 0.05 and **P < 0.01.
